# The Effect of the Strengthen Opioid Misuse Prevention Act on Opiate Prescription Practices Within the Orthopaedic Surgery Department of an Academic Medical Center

**DOI:** 10.5435/JAAOSGlobal-D-20-00006

**Published:** 2020-03-04

**Authors:** Fernando Aran, Kevin Y. Wang, Samuel Rosas, Kerry A. Danelson, Cynthia L. Emory

**Affiliations:** From the Department of Orthopaedic Surgery, Wake Forest Baptist Medical Center, Winston-Salem, NC.

## Abstract

In 2017, the Department of Health and Human Service declared a public health emergency known as the opioid crisis. In North Carolina, the “Strengthen Opioid Misuse Prevention Act of 2017” (STOP Act) went into effect on January 1, 2018, seeking to strengthen oversight over opioid prescriptions. Among other mandates, this legislation limited the duration of the initial prescription to 5 or 7 days. The purpose of this study was to compare narcotic prescription practices within the Department of Orthopaedic Surgery at an academic medical center before and after the enactment of the STOP Act. We hypothesized that there would be a statistically significant decrease in the amount of postoperative opioids prescribed after the STOP Act and that this decrease would be consistent across all types of providers in the Orthopaedic Surgery Department.

**Methods:**

Opiate prescriptions data from all orthopaedic surgery providers at our academic institution were collected from January to the end of September in 2017 and from January to the end of September in 2018. After filtering the providers by our study's inclusion and exclusion criteria, we included data from 49 providers in our analysis. We used a paired *t*-test to compare the prescription data between the two periods.

**Results:**

There was a 35% decrease in morphine milligram equivalents prescribed at our institution between 2017 and 2018 (*P* = 0.0003). This reduction was statistically significant and equaled 27,374 less morphine milligram equivalents prescribed per provider (95% confidence interval 13,226 to 41,523). The average number of opiate prescriptions per provider decreased from 171.5 in 2017 to 161 in 2018 (*P* = 0.48), although this was not statistically significant.

**Conclusion:**

The STOP Act effectively decreased the amount of opiates prescribed within our Orthopaedic Surgery Department. Similar legislation may be effective in other states and at the federal level to decrease narcotic prescriptions and subsequent abuse.

In 2017, the Department of Health and Human Service declared a public health emergency known as the opioid crisis, which was highlighted by the 2.1 million Americans reported to have drug dependence in the previous year and the 47,600 lives lost from overdose.^1^ Orthopaedic surgeons play a central role in this opioid epidemic. Among physicians, orthopaedic surgeons are the third highest opioids prescribers, accounting for an estimated 7.7% of all opioid prescriptions in the United States.^2^

Regulation of opioid prescriptions has been attempted at both clinical and legislative levels in response to the opioid epidemic. In 2016, Massachusetts was the first state to legislate opioid prescriptions by implementing a 7-day prescription limit for first-time opioid patients.^3^ Since then, many other states have enacted similar regulations. As of October 2018, 33 states have passed state legislation limiting opioid prescriptions in the postoperative period.^4^ In the state of North Carolina, the “Strengthen Opioid Misuse Prevention Act of 2017” (STOP Act) went into effect on January 1, 2018, seeking to strengthen oversight over opioid prescriptions. The STOP Act requires prescribers and pharmacies to review a patient's 12-month history before issuing an initial prescription for a schedule II or schedule III opioid, instituting a 5-day limit on initial prescriptions for acute pain and a 7-day limit on postoperative prescriptions (with exemptions for chronic pain, cancer care, palliative care, hospice care, or medication-assisted treatment of substance use disorders), while increasing access to naloxone for reversal of opioid overdose.^5^

Several studies have investigated the efficacy of various mechanisms for controlling opioid prescriptions, including the effects of specific state legislature similar to the STOP Act on opioid prescriptions in states such as Rhode Island and New York.^6,7,8,9,10,11,12^ To the authors' knowledge, however, no previous study has analyzed the effects of the STOP Act specifically on opioid prescription practices within the Orthopaedic Surgery Department of an academic medical center in North Carolina. The purpose of this study was to compare postoperative opioid prescription practices in the Orthopaedic Surgery Department of an academic medical center before and after the STOP Act. This study also compared the opioid prescription practices of orthopaedic surgeons with those of physician assistants (PAs) within the department before and after the STOP Act. We hypothesized that there would be a statistically significant decrease in the amount of postoperative opioids prescribed after the STOP Act and that this decrease would be consistent across all types of providers in the Orthopaedic Surgery Department.

## Methods

After Institutional Review Board (IRB) approval for retrospective review was granted, opiate prescriptions data from all orthopaedic surgery providers at our academic institution was collected from January to the end of September in 2017 and from January to the end of September in 2018. There were 105 providers within the Orthopaedic Surgery Department who had a specific Drug Enforcement Agency (DEA) number and prescribed narcotics during our study period. Providers who had 10 or more opiate prescriptions in both 2017 and 2018 were analyzed. Fifty-one providers met the initial inclusion criteria, of which 2 were excluded because they were more than 2 SDs away from the mean. This yielded a final analysis with 49 providers.

**Figure 1:**
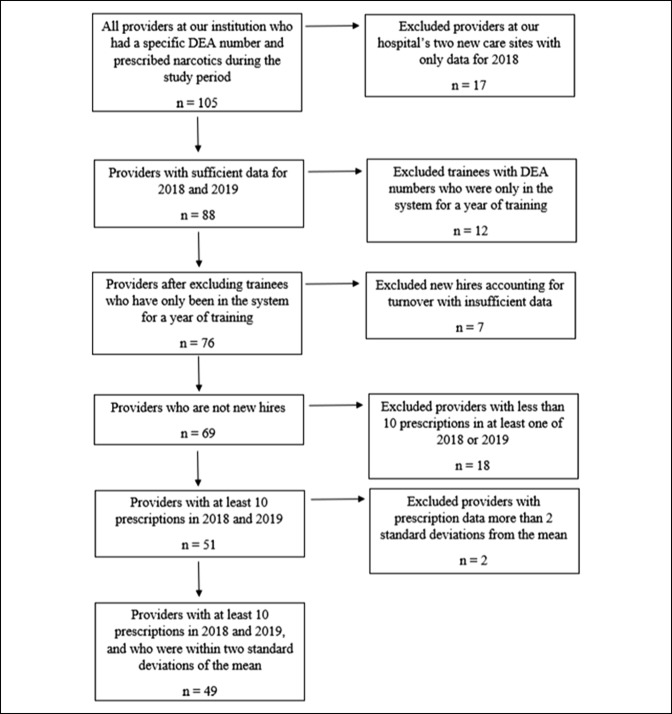
Chart illustrating providers who met the inclusion/exclusion criteria. DEA = Drug Enforcement Agency.

The large difference between the total number of orthopaedic prescribers and those who met our inclusion and exclusion criteria were primarily because of four reasons (Figure 1). First, our hospital system has acquired two new care sites and the data for these providers were only available for 2018; this accounted for 17 new providers with insufficient data to evaluate. Second, we excluded the 12 trainees with DEA numbers who were only in the system for a year of training. Third, we excluded the seven new hires because of turnover who had insufficient data to evaluate. The remaining 18 providers who we excluded had less than 10 prescriptions in at least one of the 2 years that were studied. This ultimately yielded 49 providers who met our inclusion and exclusion criteria.

After confirming a normal statistical distribution for our data, we used a paired *t*-test to analyze these 49 providers. We compared the total number of prescriptions, morphine milligram equivalents (MMEQs) prescribed, and further analyzed the type of provider by comparing the prescription data of physician assistants (PA) with the prescription data of medical doctors (MD). All data were collected and analyzed using Microsoft Excel, 2019.

## Results

There was a 35% decrease in the MMEQ prescribed at our institution between 2017 and 2018 (78,392 in 2017; 51,017 in 2018; *P* = 0.0003). This reduction was statistically significant and equaled 27,374 less MMEQs prescribed per provider (95% confidence interval 13,226 to 41,523). Converted to a number of 5 mg pills of hydrocodone, this equates to 5455 less pills per prescriber in 2018 as compared to 2017. The average number of prescriptions per provider decreased from 171.5 in 2017 to 161 in 2018 (*P* = 0.48), although this was not statistically significant (Table 1).

**Table 1:** Prescription Data for All Providers

Provider Type	Providers	MMEQ (2017)	MMEQ (2018)	Amount Reduction	95% CI	*P* Value
MD	35	77,654	49,722	27,932 (36% reduction)	12,451 to 43,413	0.0008
PA	14	80.237	54,456	25,981 (32% reduction)	−8872 to 60,834	0.1313

CI = confidence interval, MMEQ = morphine milligram equivalents, PA = physician assistant, MD = medical doctor

We further stratified our cohort by the type of prescriber, based on the data from the 35 MDs and 14 PAs included in this study. The MD cohort was responsible an average of 165 prescriptions per provider in 2017 and 148 in 2018, yielding a decrease in MMEQ of 27,932 (*P* = 0.0008), which is a 36% reduction (Table 2). Interestingly, the PA group averaged four more prescriptions in 2018 compared with their 2017 data, with a 32% decrease in MMEQ, although these were not statistically significant (*P* = 0.1313).

**Table 2:** Prescription Data Stratified by MD Versus Physician Assistant

Prescription Data	2017	2018	Amount Reduction	95% CI	*P* Value
MMEQ per prescriber	78,392	51,017	27,374 (35% reduction)	13,226 to 41,523	0.0003
5 mg pills per prescriber	15,678	10,203	5475 (35% reduction)	2645 to 8305	0.0003
Prescriptions per prescriber	172	161	11 (6% reduction)	−19 to 40	0.48

CI = confidence interval, MMEQ = Morphine milligram equivalents

## Discussion

This study shows that the implementation of the STOP Act at an academic medical center in North Carolina was associated with a notable reduction in the amount of opioid medication prescribed by the Orthopaedic Surgery Department between 2017 and 2018. Thus, our hypothesis that this study would show a statistically significant decrease in the amount of postoperative opioids prescribed after the STOP Act was validated. These findings are supported by the investigation by Reid et al^11^ on the effects of a 2017 Rhode Island law that implemented similar mandatory opioid prescription limits. Reid et al reported that the Rhode Island legislation reduced both MMEQ prescribed and the number of pills prescribed by >55% overall in their institution's orthopaedics department.

The success of the STOP Act in reducing the MMEQ of opioids prescribed at our institution may be due to several factors, including the legislation's strict limitations on opioid prescriptions and its implementation of hard stops in our emergency medical record (EMR) system. The legislation institutes a strict 5-day limit on initial prescriptions for acute pain and a 7-day limit on postoperative prescriptions, albeit with some exceptions. Previous studies have demonstrated the effectiveness of implementing prescription protocols to reduce opioid prescriptions. Earp et al^13^ showed a notable reduction in the MMEQ of opioids prescribed after the implementation of a five-tiered opioid prescription protocol that established institutional guidelines for opioid prescriptions based on the type of hand surgery a patient underwent.^13^ Although this five-tiered prescription protocol for hand surgery was established at an institutional level, rather than at a state legislative level as with the STOP Act, it suggests that the implementation of prescription limitations is generally effective at reducing opioid prescriptions. Furthermore, several studies have shown that using standardized prescription protocols reduces postoperative opioid prescriptions in many types of surgeries, not just in orthopaedic surgeries.^14,15,16,17^ Although there is currently a lack of data on the appropriate minimum quantity of opioids to prescribe after specific procedures, ultimately the literature shows that prescribing guidelines such as those implemented by the STOP Act are almost universally effective.^18^

Another contributor to the success of the STOP Act in reducing opioid prescriptions at our institution is the hard stop features implemented in our institution's EMR. These hard stops occur when providers attempt to prescribe opioids in general and when providers attempt to prescribe IV opioids when an oral alternative may suffice. When using the EMR to prescribe an opioid, a provider must check “YES” that it is indeed for the treatment of “ACUTE” pain from fracture or surgery. Only then are prescribers allowed to prescribe up to 5 or 7 days of opioids based on the dosage and frequency of the prescription. The hard stop concerning IV opioid prescriptions require the authorizing provider to give a valid reason that an oral alternative is not appropriate, thus encouraging our providers to decrease the use of IV opioid utilization on the inpatient service. These hard stops encourage providers to more intentionally consider their prescriptions, rather than prescribing out of habit. At its essence, these hard stops serve as reminders of the considerations and standards for prescribing opioids during more serious circumstances, such as for acute pain or for patients admitted to the inpatient service.

Dwyer et al^19^ showed that educating orthopaedic surgeons on recommended guidelines led to a notable reduction in opioid prescriptions, likely because of the providers being more conscious of their personal prescription practices because they were prescribing opioids to patients. The hard stops implemented by the STOP Act likely serves a similar purpose by compelling providers to be more conscious of their prescription practices and to consider prescription recommendations whenever they reach a hard stop in the EMR.

The STOP Act was effective at altering the prescription practices of both MDs and PAs in our department. At our institution, MDs saw a 36% decrease in both the number of prescriptions and total MMEQs prescribed; PAs saw a 32% decrease in total MMEQs prescribed but prescribed four more prescriptions on average after the STOP Act. The slight increase in the number of PA prescriptions may be because the PAs in our department see many of the early follow-up patients. Even so, their total MMEQs still decreased substantially.

It is important that all members of the care team endorse the same message about prescribing and using opioids for pain relief. Current literature supports a “shared mental model” that highlights the importance of all members of a care team working together to promote a unified stance on postoperative pain expectations, pain evaluation, and opioid administration.^18,20,21^ Thus, the assessment of the efficacy of an opioid control regulation must be stratified by its effect on the types of providers involved—at our institution, this includes both MDs and PAs. This study shows that the STOP Act was indeed able to decrease the MMEQ of opioids prescribed by both MDs and PAs. To the authors' knowledge, no other study has compared the effects of such legislation on different types of providers.

This study is not without limitations. It only encompasses our institution's Orthopaedic Surgery Department, so it is unknown to the study personnel if this level of success has been felt throughout all specialties. Owing to our exclusion criteria, our study may have been limited by sample size.

Further analysis of the STOP Act will be necessary to see whether the effect is sustainable at our institution and whether it has been successful at other institutions. Additional studies should be done to investigate whether the opioid prescription reduction due to the STOP Act changed how much pain patients experienced after their initial prescription, as measured by prescription refills and frequency of patient pain-related phone calls or visits to the orthopaedic clinic or emergency department. Further studies could also be done on the costs saved or incurred by our institution after the STOP Act.

## Conclusion

The STOP Act effectively decreased the amount of opioids prescribed within our Orthopaedic Surgery Department, leading to a 35% reduction in MMEQ (*P* = 0.0003) or 5455 less 5 mg hydrocodone pills per prescriber. Legislation like this may be effective in other states and at the federal level to decrease narcotic prescriptions and subsequent abuse. Ultimately, although optimal reduction in opioid prescriptions is a multimodal endeavor that is by no means limited to mere regulatory legislation, this study does demonstrate the effectiveness of the STOP Act for decreasing orthopaedic opioid prescriptions.
